# Education and training in neurology: developments and future challenges

**DOI:** 10.1111/ene.16332

**Published:** 2024-05-21

**Authors:** Matthijs van der Meulen, Maarten M. J. Wijnenga

**Affiliations:** ^1^ Department of Neurology Medisch Spectrum Twente Enschede The Netherlands; ^2^ Department of Neurology Erasmus MC Rotterdam The Netherlands

**Keywords:** education, neurology, residency, training

## Abstract

**Background and purpose:**

Training and education is essential for best practice medicine and is especially important in a rapidly evolving field such as neurology. Due to improved imaging techniques and laboratory testing, there is a better understanding of the pathophysiology of diseases. As a result more treatments have become available. The most important developments in neurology over the last two decades and their effect on training and education are described. In addition, how future training should be aware of the challenges ahead of us is described.

**Methods:**

This is a narrative review describing developments and challenges based on personal experience and the literature.

**Results:**

Due to major developments in radiological and immunological testing, major changes have been seen in different subspecialties of neurology, including but not limited to, the treatment of ischaemic stroke, the development of new entities in the field of demyelinating diseases and auto‐immune encephalitis, and diffuse glioma. These developments challenge the education and training in neurology with, ahead of us, technological developments, an aging population, and potentially more superspecialization.

**Conclusion:**

Although there are differences in the training curricula between European countries, the developments and future challenges within the field of neurology are very similar. In the development of future curricula it is important to face these developments and challenges and to adapt to them.

## INTRODUCTION

Education and training is essential for best practice medicine and for training next generation health professionals. This can be challenging especially in a rapidly evolving field such as neurology. Challenging factors are the increased complexity of our daily clinical work as neurologists due to increased understanding of the pathophysiology of many neurological diseases, discovery of new diseases, and sometimes more complex spectra within an existing disease [1, 2, 3]. Furthermore, the increasing number of diagnostic tools and therapeutic options has changed inpatient and outpatient neurological care and shifted our profession from a mostly “diagnosing and observing” specialism to a “therapeutic” specialism. This has also impacted the education and training in neurology [4].

In addition, as the prevalence of many neurological diseases rises with age and life expectancy in Europe increases, neurological care needs to expand to adequately serve the European population [5]. These factors will qualitatively and quantitatively increase the workload.

The Union Européenne des Médicins Specialistes, Section Neurology, has outlined standards for curricula of residency programs in European Union countries [6]. In addition, in 2009 the European Board Examination was initiated, which has been recognized as the end of residency official examination in some European countries. Both could help in working throughout the European Union, regardless of their training location. However, most studies evaluating the neurology curriculum for residents in European countries highlighted important educational differences [7, 8, 9]. These studies described a wide variety in duration (12–72 months) of residency training, internal (i.e., subspecialty training) and external rotations (e.g., psychiatry, neurosurgery, radiology). Despite these differences between countries, there have been major developments over the past decades that have changed the work and training in neurology across Europe. Looking to the (near) future, there are also a few challenges ahead of us. Here some important topics that were distinctive in the past years are highlighted, and future challenges for the neurology field and residency programs are identified.

## MAJOR CHANGES THAT INFLUENCED EDUCATION AND TRAINING

### Treatment of ischaemic stroke: IVT and IAT

The treatment for patients with a stroke has changed dramatically over the last decades. First, clinical care on a specialized stroke unit, with combined expertise from physicians, nurses and allied healthcare professionals, has been shown to improve survival in multiple randomized studies [10]. Second, intravenous alteplase (IVT) was introduced in 1995 for ischaemic stroke within 3 h after last seen well [11]. Subsequently, the time window of IVT was extended to 4.5 h, and later to 6 and even to 24 h [12, 13, 14]. In addition, in 2015 multiple trials showed a clinical benefit of intra‐arterial thrombectomy (IAT) in adults with a proximal occlusion of the anterior circulation [15, 16]. There is a major role for advanced imaging in identifying patients who benefit from IVT in an extended time window, with both computed tomography (CT) perfusion and magnetic resonance imaging (MRI) [17].

The developments in diagnosis and treatment of ischaemic stroke impact daily workflow and residency training. First, because of the extended time window in which IVT or IAT is possible, the team for diagnosing and treating stroke should be readily available around the clock, including neurology residents, neuro‐intervention treating physicians and support staff. Also, the frequency with which their skills are needed has increased. Second, not all centers are equipped for IAT, leading to transfer of a patient to another hospital, worsening the outcome [18]. There is a lot of debate whether the patient should be brought to a thrombectomy center in the case of a suspected stroke. This depends mostly on the regional organization of care. Regarding the residency training, it is important that those trained in a center without thrombectomy treatment options could consider having a rotation in a center that does have this possibility in order to have exposure to IAT or education about indication for IAT and about the procedure that should be incorporated in standard education for residents. Third, the interpretation of CT, CT angiography, CT perfusion, and MRI has become important in the acute setting. Although radiologists are trained to analyze imaging, also in acute care, a previous study showed that 79% of neurologists rely solely on their own interpretation when it comes to acute decision making in stroke care [19]. Meanwhile, 33% of the neurology residency curricula in the United States do not have formal training in radiology, and only 38% of the European countries have a formal rotation in radiology [9, 20]. In other words, to be able to make treatment decisions in acute stroke care, residents of neurology need to be trained in interpretation of neuro‐imaging. Lastly, interventional neurology is a subspecialty between radiology, neurosurgery and neurology, and although there is growing interest amongst neurology residents, formal training or fellowships are virtually lacking [21, 22]. All these challenges aside, considering the increasing incidence and growing treatment options, an obligatory rotation in stroke care is advocated for every resident.

### Immunology

Multiple sclerosis (MS) was and is the most well‐known demyelinating auto‐immune disease within neurology. Due to a better understanding of the pathophysiology of demyelinating diseases, a few major developments are worth mentioning. First, a new entity was recognized in 2011: a radiologically isolated syndrome (RIS), which are white matter lesions in asymptomatic patients that are found by coincidence. ‘Patients’ with RIS have a higher risk of developing MS, and it was recently shown that treatment of RIS resulted in a significantly reduced risk of developing MS [23, 24].

Second, patients can be diagnosed earlier due to changes in the diagnostic criteria of MS: with one single MRI, in the case of both enhancing and non‐enhancing lesions simultaneously since this counts as dissemination in time and space; also, by finding unique oligoclonal bands in cerebrospinal fluid (CSF) which can count as a second clinical event or MRI activity [1]. As a result of an earlier diagnosis, disease‐modifying treatment (DMT) can also be initiated earlier.

Third, within the spectrum of demyelinating diseases, a variety of new entities have been discovered. After the discovery of aquaporin‐4‐IgG (AQP4) in patients with extensive demyelinating lesions in the spinal cord and with optic neuritis, the neuromyelitis optica spectrum disorder (NMOSD) was introduced, previously called Devic syndrome. Another antibody mediated demyelinating disease is myelin‐oligodendrocyte glycoprotein antibody associated disease (MOGAD), with similar clinical and radiological findings to NMOSD, although bilateral involvement of the optic nerve is more common in MOGAD. The importance of recognizing these entities is that treatment differs from MS. Where in MS mostly DMTs are used, in the other entities B‐cell depletion therapy, such as rituximab, is used [25].

Lastly, there has been an increase in the number of available DMTs and a shift to an earlier and more aggressive treatment. DMTs can lead to an immunosuppressive state, resulting in vulnerability for infections, and nataluzimab can even lead to progressive multifocal encephalopathy [26]. Due to the developments in clinical, radiological and immunological diagnosis and in treatment strategies, knowledge of radiological interpretation and immunological assays (analysis of oligoclonal bands, AQP4 and MOG in serum and CSF) is important. Moreover, it is important to have up‐to‐date knowledge of new criteria, treatment options and their side‐effects.

A second area of immune‐mediated neurological disorders with major developments in the last 10–15 years is auto‐immune encephalitis and paraneoplastic syndromes. Antibodies that can cause neurological symptoms are not new. However, the discovery of anti‐*N*‐methyl‐d‐aspartate receptor (NMDA‐R) in 2007 accelerated the identification of many new antibodies, for example against LG1, Caspr2, AMPAR, DPPX, GABA‐A and GABA‐B receptor, each with a different association with malignancies and a different clinical and radiological presentation [27]. Early recognition of anti‐NMDA‐R encephalitis is important, since it is mainly reversible when treated early [2].

Neuro‐immunology is a subspecialty intertwined with different medical expertises, which include but are not limited to neurology, pathology, infectious disease, rheumatology, internal medicine and oncology. This might challenge education and even postgraduate fellowships in this subspeciality. The first neuro‐immunology fellowship was established in 2004 and, to date, most neuro‐immunology fellowships are still MS focused, whilst many neurological disorders, including dementia, epilepsy, neuropathies and malignancies, can be immune‐mediated. The latter is a second challenge for training and education in neurology: systemic malignancies can present with a wide variety of neurological signs and symptoms, such as movement disorders, neuropathy, ataxia or dementia [2]. So, although neuro‐immunology is an evolving subspecialty requiring in‐depth knowledge, a generalist view remains important due to the variety in clinical presentation [28].

### Genetic and molecular testing in neurology

Since the introduction of whole exome sequencing and whole genome sequencing, understanding of the pathophysiology of many neurological diseases has increased. This has led to renewed classifications and even to new entities. For instance, after the discovery of C9Orf72 in amyotrophic lateral sclerosis and frontotemporal dementia patients, C9Orf72 has been incorporated in the diagnostic criteria of these diseases [29]. Also, in an increasing number of patients with an intellectual disability and/or epilepsy a pathogenic gene was discovered due to whole genome sequencing. Within the field of pediatric neurology, gene therapy changed the life of spinal muscular atrophy patients, and the spectrum of spinocerebellar ataxias has been extended [29, 30, 31]. Within neuro‐oncology, there has been an extensive discovery and understanding of genetic and molecular changes over the last two decades. The World Health Organization classification for central nervous system tumors has been adapted twice since 2007, now combining molecular information with histological information into one integrated diagnosis. This has led to reclassification of old entities and even to new entities, which has implications for research and for informing both patients and treating physicians regarding prognosis and treatment options [3]. The challenge for education and training is that 25% of residency programs do not include neuro‐oncology, and just one European country has a voluntary rotation in clinical genetics [9]. Lastly, fluid biomarkers, such as neurofilament light chain and glial fibrillary acidic protein, have added to the understanding of many neurological diseases, such as Alzheimer's disease and small vessel disease. Although the mechanisms and cut‐off are not completely understood, they challenge again a rapidly evolving field within neurology.

### COVID‐19

The severe acute respiratory syndrome coronavirus 2 (SARS‐CoV‐2) caused a worldwide pandemic with obviously an effect on education and neurology residency training. Four major effects are described [32, 33, 34, 35]. First, education, such as lectures, grand rounds or bedside teaching, was canceled or changed into virtual sessions. The latter limits interaction and improvement of knowledge and skills [34]. Although interaction was limited in face‐to‐face education, due to the pandemic the number of online resources (e.g., webinars, e‐books and online tutorials) has increased considerably [36]. Second, outpatient visits and clinical neurophysiology examinations were canceled or reduced, which limited the volume of patients and examinations for residents, an essential component of becoming a good attending [33, 34]. Third, follow‐up visits and sometimes even new consultations were done via tele‐medicine. Residents were not trained to have these visits digitally or by telephone [32]. Lastly, many residents were reallocated to COVID‐19 wards and asked to provide care for critically ill patients with respiratory, internal medicine or even intensive care related medical problems. The latter questions whether residents should be trained in internal medicine and be equipped for acute care beyond the scope of acute neurology [32, 35]. In addition to the pandemic itself, neurological side‐effects are faced, possibly attributed to COVID‐19 vaccines, and the long‐term effects of the pandemic including neurological symptoms are still faced. Although neurological symptoms due to vaccinations or viral infections are not new, due to the scale and novelty of the COVID‐19 pandemic more neurological symptoms are faced. Since more pandemics are likely in the near future, more research is needed to better understand the neurological symptoms that can be attributed to viral infections and the vaccines against these infections [37, 38].

## CHALLENGES IN THE NEAR FUTURE

### Technological advances and artificial intelligence

In the past decade, artificial intelligence (AI) has been a key area of technological innovation. In the constantly and rapidly changing landscape of healthcare, the integration of AI into the field of neurology is a promise as it has the potential to innovate the patient journey in terms of diagnosis, treatment and prognosis. For example, it may decrease the time of diagnostic interpretation of radiological imaging and clinical neurophysiology tests (electroencephalography (EEG) and electromyography (EMG)) and could also increase the yield of it. It can aid in standardizing the application of diagnostic criteria, and integrating information across different diagnostic tests within patients. AI can aid in fine tuning existing prognostic models and in identifying new prognostic markers in large datasets. The same holds for treatment decisions, which might be more individualized with AI driven algorithms [39]. The possibilities are endless and it is almost impossible to imagine the impact on our work in the coming years.

On the negative side, the promises of AI come with challenges and pitfalls. Integration of these technologies into existing curricula without sacrificing essential clinical experience and exposure will be challenging. With the rise of AI, residents need to acquire new competencies in digital health technologies, data management and AI interpretation. The rapid pace of technological and AI revolution also requires continuous adaptation of curricula and skills. Also, there is a risk of overreliance on AI for diagnostic and therapeutic decisions, potentially undermining clinical judgment and problem‐solving skills [40].

### Increasing aging population

With an increasingly aging population, there is an anticipated increase in neurological disorders common in older adults, such as Alzheimer's disease, Parkinson's disease, stroke, and other neurodegenerative and cerebrovascular conditions [5, 41]. This will have several implications for the daily neurological practice and residency programs. The demand for neurologists specialized in age‐related disorders will rise and neurology residents may see a higher volume of patients with these conditions during their training, requiring them to develop a deeper understanding and expertise in managing complex, chronic neurological disorders. Most patients will have more comorbidities next to a single neurological disease. Although the current residency programs obviously address comorbidities, in‐depth knowledge of neurological disease is mostly focused on isolated disease whereas the clinical presentation of an elderly patient can be diverse and often encompasses more than a single illness. Therefore, future training should create awareness for the different clinical presentations of the elderly. Also, polypharmacy will become even more prevalent and knowledge about pharmacological interactions is important and requires collaboration between the neurologist, pharmacists, other medical specialties and the general practitioner.

Due to increasing age, patients with chronic neurological disease will have a longer life expectancy and thus require guidance in ‘advanced care planning’ from their primary treating neurologist. Neurology residents will probably face complex ethical dilemmas related to end‐of‐life care, decision‐making capacity and the management of chronic diseases. Training may increasingly emphasize ethical considerations, communication skills and palliative care approaches tailored to the needs of elderly patients [41].

### Decreasing number of healthcare professionals

An extra layer of complexity next to the increasingly aging population is a growing shortage of healthcare staff. A high rate of retirements reduces the number of experienced professionals available to train new staff and provide care, exacerbating the shortage. A report by the World Health Organization (2021) emphasizes the urgency of addressing the aging healthcare workforce [42]. Another significant challenge is attracting new professionals into the healthcare sector and retaining existing staff. Factors contributing to this issue include burnout, high stress levels and inadequate compensation [43]. Another factor is the challenge of medical brain drain across European countries, especially within the European Union, due to growth in migration rates of medical doctors and other healthcare professionals.

### Generalists versus superspecialists

Neurological superspecialization has emerged with highly specialized neurologists in subfractions of neurology. This is a consequence of the increased understanding of the pathophysiology of neurological diseases, with more and more (complex) treatment options available. In the context of rare neurological disorders and complex cases, superspecialization can lead to more accurate diagnoses, tailored and state‐of‐the‐art treatment plans, and better access to trials for individual patients. At the same time, especially with an increasingly aging population with comorbidities, there is a need for generalists who have broad knowledge of the general neurological signs, symptoms and disease spectrum and who can integrate neurological disease with comorbidities of other specialties. Both superspecialists and generalists play critical roles in the field of neurology, complementing each other to provide comprehensive neurological care. To address the growing demand for highly specialized neurologists, one might suggest that residents should be able to get more in‐depth training in specific areas of interest during their residency. Nonetheless, every neurologist should have a broad training base crucial particularly in outpatient clinics or when serving as the attending neurologist on call. Consequently, there is also an argument for deferring further superspecialization until after the completion of a comprehensive ‘broad neurology’ residency program [9]. This is a discussion that needs to take place, not only in the setting of neurology, but in a broader sense in the general medicine field, both nationally and internationally.

### Diversity and inclusion

In recent years there is growing awareness of disparities within the healthcare system. Diversity, equity and inclusion are more and more recognized as important aspects of medical training and in selection of medical residents, as it is important to have a healthcare workforce which represents society [44]. Future selection of candidates for neurology residency programs should focus on diversity. This is difficult, however, as the loss of diversity in the route to becoming a specialist, the so‐called leaky pipeline, starts early in life. Initiatives to improve diversity should begin prior to and during the medical school admissions process, aiming to expand the pool of potential residency candidates from underrepresented backgrounds.

Also, as this matter is not restricted only to neurology, it can be valuable to organize the approach on a national level to prevent initiatives from single institutions not leading to a desired outcome.

## SUMMARY

Over the last decades there have been major developments in many neurological diseases with regard to imaging and laboratory testing, resulting in a better understanding of pathophysiology. For some diseases this has led to new entities and more treatment options. Although these developments are of great benefit to our patients and provide opportunities in clinical care, such as AI, they come with challenges in education and training within the field of neurology, for instance keeping curricula up‐to‐date, keeping balance between generalists and superspecialists, and dealing with an aging population. This narrative review describes the most important developments and future challenges and might help in the development of future neurology education and training curricula.

## AUTHOR CONTRIBUTIONS

**Matthijs van der Meulen:** Conceptualization; writing – original draft; writing – review and editing; methodology. **Maarten M. J. Wijnenga:** Writing – original draft; writing – review and editing; methodology.

## FUNDING INFORMATION

None.

## CONFLICT OF INTEREST STATEMENT

No authors have any interest to disclose.

## Data Availability

Data sharing is not applicable to this article as no new data were created or analyzed in this study.
